# Analysis of muscle fiber conduction velocity enables reliable detection of surface EMG crosstalk during detection of nociceptive withdrawal reflexes

**DOI:** 10.1186/1471-2202-14-39

**Published:** 2013-03-26

**Authors:** Michael Brun Jensen, José Alberto Biurrun Manresa, Ken Steffen Frahm, Ole Kæseler Andersen

**Affiliations:** 1 Integrative Neuroscience group, Center for Sensory-Motor Interaction, Department of Health Science and Technology, Aalborg University, Fredrik Bajers Vej 7, E1-102, Aalborg, 9220, Denmark

**Keywords:** Nociceptive withdrawal reflex (NWR), Reflex detection, Surface electromyography (sEMG), Crosstalk, Muscle fiber conduction velocity

## Abstract

**Background:**

The nociceptive withdrawal reflex (NWR) is a polysynaptic spinal reflex that induces complex muscle synergies to withdraw a limb from a potential noxious stimulus. Several studies indicate that assessment of the NWR is a valuable objective tool in relation to investigation of various pain conditions. However, existing methodologies for NWR assessment evaluate standard surface electromyography (sEMG) measured over just one muscle and do not consider the possible interference of crosstalk originating from adjacent active muscles. The present study had two aims: firstly, to investigate to which extent the presence of crosstalk may affect NWR detection using a standardized scoring criterion (interval peak z-score) that has been validated without taking crosstalk into consideration. Secondly, to investigate whether estimation of muscle fiber conduction velocity can help identifying the propagating and non-propagating nature of genuine reflexes and crosstalk respectively, thus allowing a more valid assessment of the NWR.

**Results:**

Evaluation of interval peak z-score did apparently allow reflex detection with high sensitivity and specificity (0.96), but only if the influence of crosstalk was ignored. Distinction between genuine reflexes and crosstalk revealed that evaluation of interval peak z-score incorporating a z-score threshold of 12 was associated with poor reflex detection specificity (0.26-0.62) due to the presence of crosstalk. Two different standardized methods for estimation of muscle fiber conduction velocity were employed to demonstrate that significantly different muscle fiber conduction velocities may be estimated during genuine reflexes and crosstalk, respectively. This discriminative feature was used to develop and evaluate a novel methodology for reflex detection from sEMG that is robust with respect to crosstalk. Application of this conduction velocity analysis (CVA) entailed reflex detection with excellent sensitivity (1.00 and 1.00) and specificity (1.00 and 0.96) for the tibialis anterior and soleus muscles.

**Conclusion:**

This study investigated the negative effect of electrical crosstalk during reflex detection and revealed that the use of a previously validated scoring criterion may result in poor specificity due to crosstalk. The excellent performance of the developed methodology in the presence of crosstalk shows that assessment of muscle fiber conduction velocity allows reliable detection of EMG crosstalk during reflex detection.

## Background

The nociceptive withdrawal reflex (NWR) is a polysynaptic spinal reflex that induces complex muscle synergies to withdraw a limb from potential noxious stimuli [1]. It can be elicited by percutaneous electrical stimulation of a nerve trunk or free nerve endings located in the skin, and the resulting reflex response is often measured using standard surface electromyography (sEMG) [1,2]. Several studies indicate that assessment of the NWR is a valuable objective tool in relation to assessment of various pain conditions, which may be caused or sustained by neural hypersensitivity with a central component [3].

Several attempts have been made for standardization of NWR assessment methodologies, in order to use them as outcome measures in clinical settings. Consequently, a wide variety of definitions for NWR detection from sEMG signals has been proposed, some of which have demonstrated high accuracy and reliability [4,5]. Nevertheless, existing methodologies for NWR assessment evaluate standard sEMG measured over just one muscle and do not consider the possible interference of crosstalk originating from adjacent active muscles [1,4,5]. Electrophysiological signals are conducted through the human tissues and crosstalk may erroneously cause significant sEMG activity to be recorded over an inactive muscle [6]. As a result, some apparent reflexes may reflect nothing but crosstalk.

Crosstalk is one of the main concerns of sEMG recording, and several methods have been proposed to reduce these misleading signal components [7,8]. Unfortunately, neither high-pass temporal or spatial filtering nor cross-correlation of signals detected over different muscles has resulted in a reliable method for reduction or estimation of crosstalk [9,10]. However, it has been demonstrated that signals generated by superficial motor units of the active muscle monitored are propagating along the muscle fibers, whereas crosstalk signals are non-propagating [10]. Thus, identification of crosstalk may possibly be based on assessment of whether the signal is dominated by propagating or non-propagating signal components.

The present study had two aims: firstly, to investigate to which extent the presence of crosstalk may affect NWR detection using a standardized scoring criterion [4] that was validated without taking crosstalk into consideration. Secondly, to investigate whether estimation of muscle fiber conduction velocity (CV) can identify the propagating and non-propagating nature of genuine reflexes and crosstalk respectively, thus allowing a more valid assessment of the NWR. More specifically, it was examined if estimations of muscle fiber CV were higher for crosstalk than for genuine reflexes in the lower extremities of humans. The discriminative value of features extracted using a simple cross-correlation technique (including CV) was analyzed and a novel cross-correlation based CV analysis (CVA) for NWR detection with improved specificity is presented.

## Methods

### Participants

Fourteen male volunteers (mean age 24.4 years, range 19–28 years) participated in the study. Written informed consent was obtained from all subjects prior to participation and the Declaration of Helsinki was respected. The study was approved by the local ethical committee (‘Den Videnskabsetiske Komité for Region Nordjylland’) with approval number VN2005/2.

### Electrical stimulation

**Figure 1:**
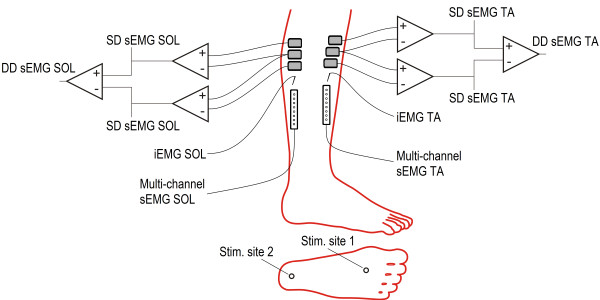
**Experimental setup.** Single differential (SD) and double differential (DD) surface electromyography (sEMG) and intramuscular electromyography (iEMG) were measured from the tibialis anterior (TA) and soleus (SOL) muscles during reflex responses elicited by electrical stimulation of two sites under the sole of the foot.

Two surface stimulation electrodes (20 × 15 mm, type 700, Ambu, Denmark) were mounted on the plantar side of the foot to elicit the NWR, as seen in Figure 1. One large common anode (100 × 140 mm, Pals, Axelgaard, USA) was placed on the dorsum of the foot to ensure that the stimulus was perceived as coming from the sole of the foot. The stimulation electrodes were moved slightly in case the evoked sensation indicated direct nerve trunk stimulation (i.e. radiating sensation). Each stimulus consisted of a constant current pulse train of five individual 1 ms pulses delivered at 200 Hz (felt as a single stimulus) by a computer controlled electrical stimulator (Noxitest IES 230, Aalborg, Denmark). The stimulation intensity was set as 1.2-1.5 times the initial NWR threshold for each electrode. The two stimulation sites were stimulated in a blinded random sequence with an inter-stimulus interval between 10 and 15 s.

### EMG recordings

Activity in the ipsilateral tibialis anterior (TA) and soleus (SOL) (lateral side) was measured using single-channel and multi-channel sEMG and also intramuscular electromyography (iEMG), see Figure 1 for complete setup.

#### Single-channel sEMG

Three surface electrodes (type 720, Ambu, Denmark) were placed in parallel along the overall orientation of the two muscles with an interelectrode distance of 2 cm. A common reference electrode (100 × 140 mm, Pals, Axelgaard, USA) was placed on the ipsilateral knee. The tri-polar electrode configuration fed three separate amplifiers for simultaneous recording of two single differential (SD) and one double differential (DD) sEMG signal from each of the two muscles, see Figure 1. The signals were amplified, filtered (5–500 Hz), sampled (2 kHz) and stored (1 s window including 200 ms pre-stimulation). The signals were interpolated to 10 kHz using an antialiasing, linear-phase, low-pass, finite impulse response filter implemented in Matlab (MathWorks, USA).

#### Multi-channel sEMG

Multi-channel sEMG signals were detected with two linear adhesive arrays (type ELSCH008, Spes Medica, Italy), each consisting of eight electrodes with 5 mm inter-electrode distance in bipolar configuration. The adhesive arrays were placed along the overall orientation of the muscles just proximal to the distal tendon. The signals were amplified (EMG-USB2, OT Bioelettronica, Torino, Italy), filtered (10–500 Hz) and sampled at 10 kHz.

#### iEMG

iEMG signals were recorded using wire electrodes made of teflon-coated stainless-steel (50 μm diameter, A-M Systems, Inc. Carlsborg, USA) with 5 mm un-insulated tips in bipolar configuration [11,12]. The skin was cleaned with alcohol at the insertion points whereupon wire electrodes were placed inside each of the two muscles using a concentric hypodermic needle (25 gauge, 25 mm). The signals were amplified (EMGUSB2, OT Bioelettronica, Torino, Italy), filtered (10–500 Hz) and sampled at 10 kHz.

### Experimental procedure

Before mounting the recording and stimulation electrodes, thick epidermal layers on the sole of the foot were ground off using a callus remover and the skin was scrubbed with an abrasive paste in order to reduce skin impedance. The skin above the two muscles was shaved and slightly abraded with an abrasive paste. The subject was sitting relaxed on a chair with the hip, knee and ankle flexed 90 degrees. The subject was thoroughly familiarized with electrical stimulation before actual data acquisition. The NWR threshold was identified for each of the two stimulation sites as the lowest intensity eliciting at least two reflexes in three consecutive stimulations. The subjects were stimulated until ten unambiguous reflexes (see Identification of genuine reflexes) had been recorded from both the TA (stimulation in the arch of the foot) and SOL (stimulation at the heel) muscles. If elicitation of reflexes ceased at a site during the experiment e.g. due to habituation (no reflex elicited by three consecutive stimulations of one site), the NWR threshold for that stimulation site was re-assessed and an adjusted stimulation intensity was applied. If the stimulation intensities became intolerable to the subject the experiment was discontinued.

### Data analysis

#### Identification of genuine reflexes

**Figure 2:**
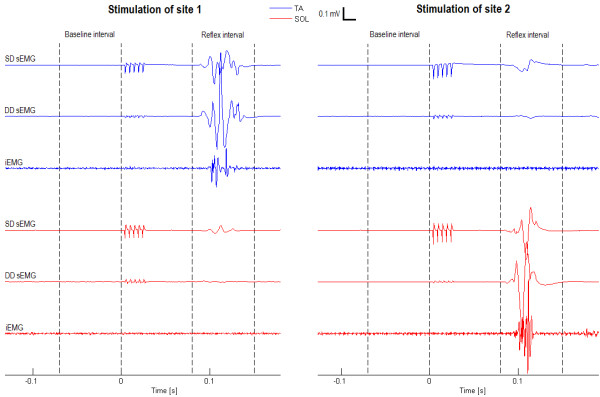
**EMG signals recorded during elicited reflexes.** Single differential (SD) and double differential (DD) surface electromyography (sEMG) and intramuscular electromyography (iEMG) signals recorded during a genuine reflex response involving activity in either the tibialis anterior (TA) or the soleus (SOL) muscle exclusively. When evaluating SD sEMG only, both visual inspection following the fixed criteria proposed in [4] and automated evaluation of a standardized scoring criterion (interval peak z-score) indicate reflex responses involving both muscles. However, the DD sEMG and iEMG signals reveal that genuine muscle activity is present in only one of the two muscles depending on the stimulation site.

During data acquisition, the recorded signals were visually examined for online identification of NWR using a set of fixed criteria proposed by Rhudy and France [4], i.e., a reflex was identified if at least one sizable difference peak occurred in the reflex window (defined as the 80–150 ms post-stimulus interval), relative to baseline, but not if EMG activity in the reflex window mimicked baseline, see Figure 2. Additional criteria involving the simultaneous assessment of sEMG and iEMG signals were enforced post acquisition to ensure a homogenous dataset, consisting of sweeps containing a genuine reflex in only one of the two antagonistic muscles for data analysis.

The inclusion and exclusion criteria can be summarized as follows. *Inclusion*: *(1)* Reflex identified in all sEMG recordings and a simultaneous reflecting burst of iEMG activity recorded from the same muscle (any concurrent sEMG activity measured over the antagonistic muscle was considered crosstalk), see Figure 2. *Exclusion: (1)* EMG activity in a 200 ms prestimulus window. *(2)* sEMG activity not synchronized with iEMG recordings from one of the two muscles. *(3)* iEMG activity in both muscles within the reflex window (co-contraction).

The amplitude of the NWR in the retained sweeps was quantified as the average root mean square amplitude of the two SD signals calculated within in the reflex window. The amplitude of crosstalk was calculated in the same way, based on the two SD signals recorded over the non-active muscle.

#### Estimation of muscle fiber conduction velocity

Muscle fiber CV was estimated from multi-channel sEMG recordings of sweeps using a maximum likelihood estimator [13]. In order to avoid unreliable CV estimations, only recordings with a signal-to-noise ratio (SNR) larger than 7 dB were included in the analysis. Additionally, a simple cross-correlation technique was applied on the standard single-channel sEMG signals [14,15]. For each muscle, the cross-correlation between the proximal and distal SD sEMG signals was calculated and the conduction time between the two electrode pairs was estimated as the temporal displacement of the peak in the cross-correlogram. Average CV was calculated as the inter-electrode distance divided by the estimated conduction time. Accurate estimation of average CV requires undistorted propagation of the signals along the muscle fibers. While this ideal assumption is never completely valid in practice due to the finite length of fibers, violations are particularly prone to occur for signal components with long wavelengths relative to the actual length of the muscle fibers [16]. Hence, prior to performing the cross-correlation, the SD sEMG recorded using single-channel electrodes over TA and SOL were high-pass filtered with cut-off frequencies of 80 Hz and 100 Hz, respectively.

#### Reflex detection by evaluation of interval peak z-score

The SD and DD sEMG signals were rectified and their interval peak z-score [4] was calculated as:

(1) $$\mathrm{Interval}\;\mathrm{peak}\;\mathrm{z-score}\;=\;\left(\mathrm{reflex}\;\mathrm{window}\;\mathrm{peak}\;-\;\mathrm{baseline}\right)/\mathrm{baseline}\;\mathrm{standard}\;\mathrm{deviation}$$

The literature suggests that signals with an interval peak z-score larger than 12 represent a reflex [5]. The sensitivity (i.e. the ability to detect genuine reflexes) and specificity (i.e. the ability to avoid detecting crosstalk as genuine reflexes) of this approach were investigated. Two different visual examinations of the recorded signals were used for validation, considering sEMG and iEMG signals respectively.

#### Cross-correlation based CVA for reflex detection

This novel method for reflex detection involved analysis of cross-correlations between the two SD sEMG signals recorded over each muscle and evaluation of features extracted from the resulting cross-correlograms. The cross-correlations were normalized by the product of the norm of each of the two SD sEMG signals.

**Figure 3:**
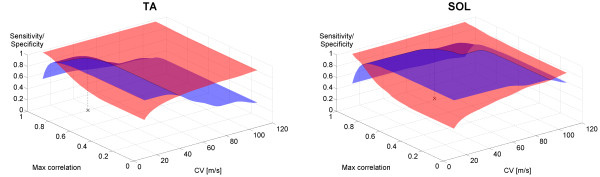
**Sensitivity (red/light) and probability (blue/dark) for cross-correlation based conduction velocity analysis (CVA).** This novel methodology for reflex detection is based on evaluation of muscle fiber conduction velocity (CV) and maximal cross-correlation estimated from surface electromyography. Sensitivity and specificity is presented for both the tibialis anterior (TA) and soleus (SOL) muscles. The black cross indicates the identified thresholds entailing an equally weighted joint value for sensitivity and specificity of 0.96 and 0.91 for TA and SOL respectively.

The method was designed to determine if a response associated with an interval peak z-score larger than 12 indeed represents a genuine reflex or may be attributed to crosstalk. Hence, it was only applied on recordings where both SD sEMG recordings and the DD sEMG recording were associated with an interval peak z-score larger than 12 (otherwise no reflex was detected). The response were attributed to crosstalk (and no reflex was detected) if both the CV estimated from the cross-correlation and the maximal value of the normalized cross-correlation were above fixed thresholds (specific for each muscle). The thresholds for CV and maximal correlation were identified by simultaneous optimization of both sensitivity and specificity based on pooled data from all sweeps, i.e., maximization of the intersection between the sensitivity and specificity planes in Figure 3.

#### Comparison of methods for reflex detection

CVA was compared to reflex detection based on evaluation of interval peak z-score calculated for both SD and DD sEMG signals by evaluation of sensitivity and specificity calculated for each individual subject.

### Statistics

Parametric and non-parametric statistical methods were used whenever data was normally or non-normally distributed, respectively. Mann–Whitney *U* test was used for comparison of CV, whereas Friedman test was used for paired comparison of both sensitivity and specificity of methods for reflex detection, with Student Newman Keuls for post hoc analysis. Independent t-tests were used for comparison of amplitudes of reflexes and crosstalk, respectively. P < 0.05 was considered statistical significant. Results are presented as mean ± standard error of the mean when the underlying data is normally distributed and as median (lower quartile, upper quartile) when it is not.

## Results

The initial reflex thresholds for stimulation of the arch and heel were 9.5 ± 1.4 mA and 14.9 ± 1.3 mA, entailing initial stimulation intensities of 12.6 ± 1.6 mA and 20.4 ± 1.7 mA, respectively. The amplitudes of reflexes elicited in TA (91.8 ± 3.9 μV) were significantly larger than those elicited in SOL (34.0 ± 2.3 μV) (t_(487)_ = 10.76, p < 0.001) and consequently the amplitudes of crosstalk measured over SOL (8.2 ± 0.6 μV) were significantly larger than those measured over TA (6.7 ± 0.3 μV) (t_(487)_ = 13.47, p < 0.001). The mean number of genuine reflexes identified in each subject in TA and SOL was 22.0 (range 5–36) and 12.9 (range 5–23), respectively. In comparison, the mean number of reflexes detected by evaluation of interval peak z-scores (including genuine reflexes and crosstalk erroneously detected as reflexes) was 30.5 and 28.5 in TA and SOL, respectively.

### Muscle fiber conduction velocity

Due to high selectivity of the multi-channel array electrodes, only 7 and 36 cases of crosstalk (in 308 and 181 recorded reflexes) with a SNR larger than 7 dB were measured over TA and SOL, respectively. However, muscle fiber CV estimated from both multi-channel sEMG (both muscles, U_(7,36)_ = 0, z = 4.13, p < 0.001) and from sEMG recorded using tri-polar configurations of single-channel electrodes (TA: U_(308,119)_ = 2603, z = 13.76, p < 0.001; SOL: U_(181,219)_ = 2557, z = 15.09, p < 0.001) clearly demonstrated that crosstalk was associated with significantly higher estimations of muscle fiber CV than genuine reflexes for both muscles, see Table 1.

**Table 1:** **Muscle fiber conduction velocity (CV) estimated from multi-channel surface electromyography (sEMG) using a maximum likelihood estimator **[13]**and from two single differential sEMG recordings using a simple cross-correlation technique **[14,15]**, respectively**

**CV [m/s]**		**Reflex:**	**Crosstalk:**
*Maximum likelihood estimator*	**TA:**	6.0 (5.0-7.1)	168.7 (93.0-198.3)
	**SOL:**	7.8 (6.8-9.9)	90.1 (53.6-146.5)
*Simple cross-correlation technique*	**TA:**	5.3 (2.8-14.3)	66.7 (50.0-100.0)
	**SOL:**	8.7 (4.2-40.0)	200.0 (100.0-200.0)

### Reflex detection by evaluation of interval peak z-score

**Figure 4:**
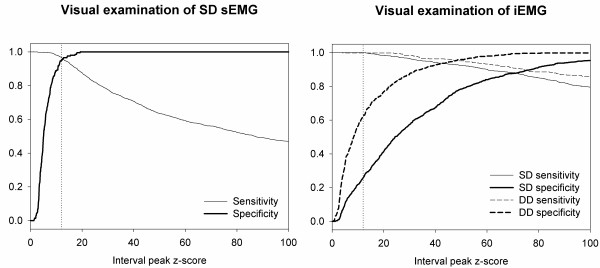
**Sensitivity and specificity of reflex detection based on evaluation of interval peak z-scores.** The sensitivity and specificity was calculated using two different gold standards; visual examination of single differential (SD) surface electromyography (sEMG) and intramuscular electromyography (iEMG) recordings respectively. The vertical lines represent an interval peak z-score threshold of 12.

The evaluation of interval peak z-score entailed reflex detection with high sensitivity and specificity, when it was validated using solely visual examination of SD sEMG. Moreover, in line with previous studies [4,5], the optimal threshold for the interval peak z-score was 12.6 (at the intersection of sensitivity and specificity curves) assuming equal cost functions for sensitivity and specificity, see Figure 4. In contrast, the use of visual examination of iEMG recordings for validation (allowing distinction between genuine reflexes and crosstalk) indicated that the optimal thresholds for the interval peak z-score were 71 and 48 for evaluation of SD and DD signals respectively, see Figure 4. This comparison furthermore demonstrated that application of an interval peak z-score threshold of 12 was associated with poor reflex detection specificity (0.26 and 0.62 respectively) for SD and DD signals.

### Cross-correlation based CVA for reflex detection

The thresholds for CV for TA and SOL were 34 m/s and 68 m/s, whereas the thresholds for maximal cross-correlation were 0.80 and 0.82, respectively. These thresholds are indicated in the sensitivity and specificity plots in Figure 3, which illustrates the discriminative power of muscle fiber CV. Evaluation of CV alone (intersection with the CV-axes in Figure 3) allows an equally-weighted joint value of sensitivity and specificity of 0.90 for both TA and SOL. Moreover, adding the maximal cross-correlation feature results in an increase in joint sensitivity and specificity to 0.96 and 0.91 for TA and SOL, respectively.

### Comparison of methods for reflex detection

**Figure 5:**
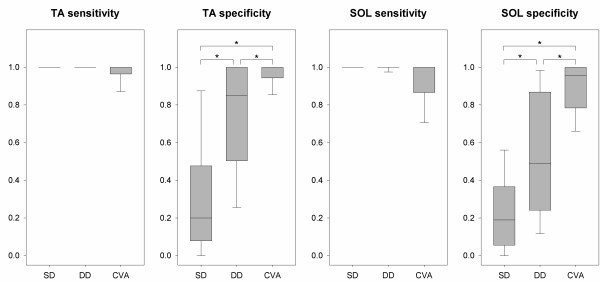
**Comparison of sensitivity and specificity of the applied methods for reflex detection.** Reflex detection based on evaluation of interval peak z-scores, performed on both single differential (SD) and double differential (DD) surface electromyography signals, where compared to cross-correlation based conduction velocity analysis (CVA). The plots display the sensitivity/specificity calculated for each individual subject for both the tibialis anterior (TA) and soleus (SOL) muscle. Asterisks indicate significant post hoc pairwise comparisons.

CVA showed a significant increase in specificity compared to reflex detection by evaluation of interval peak z-score performed on both SD and DD sEMG signals, see Figure 5. However, reflex detection performed on SD sEMG had significantly lower specificity than detection performed on DD sEMG (TA: *x*^2^_(2)_ = 26.08, p < 0.001; SOL: *x*^2^_(2)_ = 25.56, p < 0.001; all post hoc pairwise comparisons: p < 0.05). A significant difference was identified regarding the sensitivity of the three detection methods, meaning that they are not likely to present the same median sensitivity (TA: *x*^2^_(2)_ = 10.00, p = 0.007; SOL: *x*^2^_(2)_ = 9.50, p = 0.009). However, pairwise significant differences were not found, likely due to lack of power of the post hoc multiple comparison tests (no significant post hoc pairwise comparisons: p > 0.05).

## Discussion

This study investigated the negative effect of electrical crosstalk during reflex detection and revealed that the use of a previously validated scoring criterion may result in poor specificity in the presence of crosstalk. Two different standardized methods for estimation of muscle fiber CV were employed to demonstrate that significantly different CVs may be estimated during genuine reflexes and crosstalk, respectively. This discriminative feature was used to develop and assess a novel methodology for reflex detection from sEMG that is robust with respect to crosstalk.

### Identification of genuine reflexes

The sEMG and iEMG signals recorded together with the set of fixed criteria described in the Methods section, based on physiological knowledge of the human withdrawal reflex, clearly indicate that the signals regarded as crosstalk are indeed so. This conclusion can mainly be drawn due to temporal observations. Studies in both animals [17-20] and humans [21,22] have demonstrated that nociceptive withdrawal reflexes are modularly organized, meaning that each muscle or group of synergistic muscles has a bounded well-defined and unique cutaneous reflex receptive field (RRF). Noxious stimulation of the skin within the RRF may cause a reflex response involving the related muscles, whereas stimulation outside the RRF has no effect or may even inhibit activity in the same muscles [18,19,23]. The RRF is hence defined as the skin area from which a reflex can be evoked, which generally adheres to biomechanical function of the related muscle ensuring efficient withdrawal [17,19]. During voluntary contraction, the two antagonistic muscles may likely be activated simultaneously (co-contraction) to stabilize a joint. However, due to the functional modular organization of the NWR, this is highly unlikely to occur during a reflex response. A few times during the data acquisition, activity in both muscles were observed within the reflex window. However, these occurrences were never synchronized in the recordings from the two muscles, but could be synchronically identified in both the sEMG and iEMG signals recorded from each of the two muscles, respectively. These recordings were not included in the data analysis, in accordance with the exclusion criteria.

### Validation of automated scoring criteria

Several automated scoring criteria, including the interval peak z-score, have been demonstrated to enable accurate and reliable reflex detection from sEMG signals [4,5]. This comprehensive validation of scoring criteria contributed to the standardization of reflex detection methodology and promoted the evaluation of reflex thresholds as a valuable experimental and clinical tool. However, the validation performed did not consider EMG crosstalk, and the applied gold standard (visual examination of the sEMG signals), did not allow a proper evaluation of the authenticity of any apparent reflex observed as illustrated in Figure 2.

The present study utilized iEMG to obtain an improved gold standard that allows for a distinction between crosstalk and genuine reflexes. The results demonstrated that crosstalk may easily fulfill both the investigated scoring criteria (interval peak z-score > 12) and the criteria for subjective assessment presented by Rhudy and France [4] and France et al. [5]. Consequently, the validation of the interval peak z-score seems limited to the ability to detect ongoing electrophysiological activity. The origin of the electrical signal (the effectuating muscles) is not considered, rendering muscle-specific reflex detection problematic.

### Crosstalk implications on reflex detection

The NWR utilizes complex muscle synergies to effectively withdraw a limb from potential noxious stimuli and will often involve activation of more than one muscle [1]. This may cause a sEMG recording of a reflex response to be a mixture of signals originating from various muscles. Consequently, valid muscle specific reflex detection requires a distinction between signal components originating from adjacent muscles (i.e. crosstalk) and genuine muscle activity in the investigated muscle. This is of particular importance whenever the muscles executing the reflex response are of interest e.g. during mapping of RRF or assessment of RRF modulation for specific muscles in relation to variation in nociceptive sensitivity [22].

This study clearly demonstrated the possible consequences of disregarding crosstalk during reflex detection. The evaluation of interval peak z-scores calculated for SD sEMG signals (the standard recording of reflexes in both experimental and clinical settings) resulted in reflex detection with an extremely poor specificity (0.20 and 0.19 for TA and SOL respectively). In this study, nearly half of all detected reflexes were not genuine reflexes but merely reflecting crosstalk. The amount of crosstalk depends on anatomical conditions, such as thickness of the subcutaneous layer [7,8], and clearly on the magnitude of the originating muscle activity.

The amplitude of reflexes in the present study may be modulated in several ways, most effectively by manipulation of stimulation intensities. Consequently, the specificity for reflex detections based on evaluation of interval peak z-scores may be influenced by varying stimulation intensities. However, the stimulation intensities applied in this study (1.2-1.5 times the reflex threshold) were lower than those generally applied in previous studies using similar experimental setups [22,24]. This indicates that the magnitude of crosstalk observed in this study may be representative for NWR assessment using a similar experimental setup and hence that, under certain conditions, crosstalk may pose a serious problem during NWR assessment.

### Distinction between crosstalk and genuine muscle activity

This study has clearly demonstrated that significantly different muscle fiber CVs may be estimated for genuine reflexes and crosstalk, respectively. The underlying hypothesis has been tested using two standardized methods for CVs estimation based on different types of sEMG. The apparent CVs estimated for crosstalk are unreasonable high from a physiological perspective, reflecting that the main components of the signals are not propagating at all but are observed roughly simultaneously at the two adjacent recording sites. The apparent CVs estimated due to electrical crosstalk alone were more than one order of magnitude higher than CVs estimated for genuine reflexes, rendering the precision of the simple cross-correlation technique sufficient to allow distinction between crosstalk and genuine reflexes. However, the limited precision of the cross-correlation technique and the resulting relatively large variability in CVs estimations did entail detection thresholds for muscle fiber CVs well above the physiological range.

Restrictive inclusion criteria for the individual sweeps recorded has been employed to ensure the existence of two separate datasets consisting exclusively of crosstalk and genuine reflexes, respectively. While appropriate and necessary in order to properly demonstrate the differences in CV for crosstalk and genuine muscle activity, it leaves room for more complex scenarios, involving both crosstalk and genuine reflex activity within the reflex window for further research. When practical measures are considered, crosstalk may be due to a combination of travelling and non-travelling signal components depending on the distance between the active muscle fibers and the detection point [8]. In the case of a mixture of crosstalk and genuine muscle activity an estimation of CV will involve a weighted average of the temporal delay of both propagating and non-propagating signal components originating from both crosstalk and genuine muscle activity. Whether estimation of CV of a recorded signal indeed reflects the amount of crosstalk in a reliable manner needs to be investigated and could potentially allow not only detection but also estimation of crosstalk during reflex detection.

### Conduction velocity estimation

Various CV estimation techniques differ with respect to specific definitions of the delay between signals which in practice have unequal shapes [25]. Hence, minor discrepancies between CVs estimated using different methods are inevitable. However, the most evident difference between the two sets of muscle fiber CV estimations is the width of their confidence intervals. CV estimation using a sophisticated algorithm performed on several sEMG channels recorded using an electrode array had a higher precision than the much simpler cross-correlation technique performed on two SD sEMG channels. This was expected and the precise maximum likelihood estimator was intended for validation of estimated CVs and to constitute reference values for future work.

Reliable evaluation of whether a signal is dominated by propagating or non-propagating signals components requires estimation of CV with a certain degree of accuracy and precision. The simple and convenient method for muscle fiber CV estimation, constituting the core of this new methodology for reflex detection, is best suited for long superficial muscles with parallel fibers like TA. The performance of the novel methodology may therefore vary when applied on different muscles, but even evaluation of a bi-pennate muscle with short, non-parallel fibers like SOL did allow reflex detection with excellent accuracy.

Whereas the CVs estimated for TA were slightly higher than previous findings [16], the CVs estimated for SOL in the present study were definitely higher than physiologically reasonable. However, a considerable overestimation had to be expected considering the rather wide pennation angle for this muscle. SOL has a pennation angle of approximately 25 degrees at rest [26], reducing the effective inter-electrode distance along the orientation of the fibers about 10%, causing an equivalent overestimation of the average CV.

Furthermore, Broman et al. [27] reported CVs up to 8 m/s for TA estimated using the applied cross-correlation technique on SD signals, whereas application of the same technique on DD recordings eliminated these supra-physiological CV estimations. These high CVs may be caused by the existence of both propagating and non-propagating signal components due to inhomogeneity and anisotropic properties of the volume conductor and may possibly be reduced by evaluation of cross-correlations of DD signals instead of SD recording.

Additionally, the high-pass filtering prior to CV estimation using the cross-correlation technique may result in CV overestimation. The cut-off frequencies constitute a compromise between maintenance of a sufficient high SNR and rejection of distorted waves, and will accordingly differ between the two muscles due to marked difference in muscle fiber length. As such, an optimal cut-off frequency cannot be selected, especially for SOL. The applied cut-off frequency at 100 Hz attenuates signal components with wavelengths exceeding 40 mm considering an average CV of 4 m/s, whereas the length of SOL muscle fibers at rest are approximately 35–38 mm [26]. Even under the most unlikely assumption (that the motor end plates are located at the end of the fibers), distorted waves remain. However, increasing the cut-off frequency will result in a SNR that would be too low to allow meaningful CV estimation.

### Applicability and necessity of improved reflex detection

The validation of the interval peak z-score, based on visual examination of SD sEMG recordings, carried out in this study supports previous findings [4,5]; a threshold value around 12 will allow accurate and reliable detection of apparent reflexes. However, in the presence of crosstalk, not all electrophysiological activity observed represents a genuine reflex involving the muscle investigated. This potential issue was elucidated by the application of a refined gold standard (visual examination of iEMG [28]), allowing distinction between crosstalk and genuine reflexes and resulting in improved validation. It was hereby revealed that application of an interval peak z-score threshold of 12 to achieve muscle specific reflex detection may result in an extremely poor specificity, especially when performed on SD sEMG signals. As shown in the plots of sensitivity and specificity (Figure 4), a strikingly improved specificity combined with a reasonable sensitivity could be achieved by setting a much higher threshold for the interval peak z-score. Evaluation of interval peak z-scores calculated for DD sEMG signals may allow a joint value of sensitivity and specificity of 0.95 if the threshold was set at 48 instead of 12.

It is nevertheless stressed that one optimal, fixed threshold for the interval peak z-score cannot be established, and that custom thresholds should be chosen with great care. The application of a very high threshold in order to distinguish genuine reflexes from crosstalk based on the magnitude of the electrophysiological measurements would work well on this specific dataset. However, this will probably not be the general case. Both the optimal interval peak z-score threshold and the resulting sensitivity and specificity may vary strongly depending on the data in question. In any case, sufficiently small reflexes will be mistaken for crosstalk and erroneously undetected. Since experimental and clinical protocols often emphasize evaluation of reflex thresholds, this poses a serious problem. This problem does not arise when applying CVA for reflex detection, which constitutes a major advantage of this novel methodology.

The optimal method for reflex detection depends on specific challenges and requirements, including the presence of crosstalk and also weighting of sensitivity and specificity. Reflex detection based on evaluation of interval peak z-scores performed on both SD and DD sEMG entailed perfect sensitivity, indicating great performance in the absence of crosstalk. However, in the presence of crosstalk, the evaluation of DD sEMG instead of SD signals may entail a significant improvement in detection accuracy. This is clear from the plots of sensitivity and specificity (Figure 4) where both sensitivity and specificity, for all interval peak z-score thresholds, are superior for evaluation of DD sEMG compared to SD sEMG. The statistical analysis and the box-plots in Figure 5 suggest that CVA seems to entail slightly lowered sensitivity, especially for SOL. Thus, the relative value of sensitivity and specificity respectively ought to be weighted prior to deciding whether to apply the novel methodology or to purely evaluate interval peak z-scores calculated for DD sEMG. Also the risk and magnitude of crosstalk should be considered. In cases with seldom and weak crosstalk, the specificity achieved by reflex detection based on evaluation of interval peak z-scores calculated for DD sEMG may be sufficient, rendering superior sensitivity. However, whenever muscle specific reflex detection with a reliable high specificity is required, CVA should be seriously considered.

### Beyond reflex detection

CVA may be viewed as an additional binary evaluation following another reflex detection methodology, in order to assess whether a detected reflex indeed is a genuine reflex or merely the result of crosstalk. There seems to be no reason why this approach should be less efficient detecting crosstalk during static or voluntary contractions. Hence, this paper presents a convenient generic method for qualitative assessment of crosstalk, applicable on signals recorded using standard sEMG equipment and procedures which may possibly be utilized to ensure a more specific and reliable detection of genuine muscle activation e.g. during gait analysis, biofeedback therapy, prosthetic control, or other applications.

## Conclusion

This study demonstrated possible consequences of disregarding electrical crosstalk during reflex detection from sEMG signals. Evaluation using a previously validated scoring criterion (interval peak z-score) calculated for standard SD sEMG signals resulted in reflex detection with poor specificity due to crosstalk. In the presence of crosstalk, the evaluation of DD sEMG instead of SD signals may entail a significant improvement in detection accuracy. Furthermore, this study demonstrated that significantly different muscle fiber CVs may be estimated for genuine reflexes and crosstalk, respectively. A novel methodology, CVA, was developed to allow reliable detection of EMG crosstalk during reflex detection, which resulted in reflex detection with excellent sensitivity and specificity.

## Abbreviations

CV: Conduction velocity; CVA: Conduction velocity analysis; DD: Double differential; iEMG: Intramuscular electromyography; NWR: Nociceptive withdrawal reflex; RRF: Reflex receptive field; SD: Single differential; sEMG: Surface electromyography; SNR: Signal-to-noise ratio; SOL: Soleus; TA: Tibialis anterior.

## Competing interests

The authors declare that they have no competing interests.

## Authors’ contributions

MBJ, JBM and OKA defined the research topic. MBJ and KSF designed the experiment and carried out the data acquisition. MBJ analyzed the data, developed the presented methodology, interpreted the results and wrote the paper. JBM and OKA have contributed to the analysis, interpretation, and presentation of the paper. All authors have read and approved the final manuscript.
